# Application and challenge of Schwann cell transplantation in spinal cord injury and clinical trial

**DOI:** 10.1097/JS9.0000000000002955

**Published:** 2025-07-31

**Authors:** Mu-fa Lu, Ji-peng Liu, Yong-sheng Xu, Cheng Zuo, Si-cheng Liu, Wen-jun Zhang

**Affiliations:** a Department of Emergency, The Second Affiliated Hospital, Jiangxi Medical College, Nanchang University, Nanchang, Jiangxi, China; b Department of Gastrointestinal Surgery, The Second Affiliated Hospital, Jiangxi Medical College, Nanchang University, Nanchang, Jiangxi, China; c Department of Rehabilitation Medicine, The Second Affiliated Hospital, Jiangxi Medical College, Nanchang University, Nanchang, Jiangxi, China

**Keywords:** clinical trials, mechanism, Schwann cells (SCs), spinal cord injury (SCI), transplantation

## Abstract

Spinal cord injury (SCI) can lead to sensory, motor, and autonomic dysfunction, and even neuropathic pain, seriously affecting the physical and mental health of patients and bringing a huge financial burden to society and families. In recent years, cell transplantation technology has rapidly entered people’s horizons and has been recognized by different studies in the field of tissue repair and regeneration. Schwann cells (SCs) are a type of glial cells that make up peripheral nerves. Their transplantation can promote SCI repair and functional recovery. The functional mechanisms of SCs as cell replacement therapy for repairing SCI include promoting axon regeneration and myelination, secreting multiple neurotrophic factors for neuroprotection, immunoregulation, and anti-inflammation, and inhibiting glial scar formation. In addition, cell combination therapy technology includes combining other types of cells or biomaterials to enhance the activity and function of SCs, which can produce synergistic therapeutic effects in SCI treatment. In clinical trials, SC transplantation is a safe and feasible treatment strategy and has certain therapeutic effects on patients with SCI. SC transplantation can improve some sensory, motor, and autonomic nervous functions of patients. However, SCs are currently in the preliminary exploration stage of clinical trials, and there are many problems and challenges in the extensive development and application of clinical trials. Therefore, here, we fully discussed the current functional mechanism of SCs in SCI repair, as well as the current status and challenges in clinical trials.

## Introduction

The pathological mechanism of spinal cord injury (SCI) is complex, which can be derived from a variety of factors, such as trauma, entrapment, immune injury, inflammation, and tumor destruction. These factors lead to varying degrees of sensory and motor dysfunction. Even cause autonomic dysfunction, including bladder function and respiratory function, with the progress of the disease can cause stubborn pathological pain. It has brought a huge burden to the patient’s family and society^[1,2]^. The key to SCI lies in neuronal degeneration/apoptosis, axon demyelination, retraction, and cleavage. The secondary changes associated with the progression of SCI include a series of pathological changes, mainly due to the changes in local microenvironment, including activation and infiltration of inflammatory cells, release of pro-inflammatory cytokines (CKs), and glial scar formation. These pathological changes lead to/aggravate ischemia, edema, and cell death^[3,4]^. These factors form a catastrophic microenvironment that is not conducive to the repair and regeneration of SCI and hinder regeneration and functional recovery. Therefore, patients need timely surgery or other treatments (such as electrical stimulation, drug intervention, including nutritional neurology drugs, functional training, and neural rehabilitation) to promote neuronal reorganization and functional compensation^[5–7]^. Surgical treatment can provide emergency surgery to relieve spinal cord compression, such as discectomy, hematoma clearance, pedicle screws, laminectomy, pedicle resection, and implantation of artificial vertebral bodies or bone graft fusion. These surgeries are effective in stabilizing the spine, thereby reducing nerve pressure, promoting recovery, and helping patients restore some neurological functions and quality of life^[1,8]^. However, surgical treatment has its own limitations, including inability to reverse nerve damage, timing of surgery [early surgery (<24 h) may reduce secondary injuries, but some patients cannot tolerate emergency surgery due to general conditions (such as shock and multiple organ damage), delay optimal intervention time], postoperative complications, and functional recovery bottlenecks. Surgery also cannot reverse the pathological changes of SCI, especially secondary pathological changes such as ischemia, inflammatory response, apoptosis/death of nerve cells, and ruptured axons because functional recovery after SCI depends on the plasticity of residual nerve tissue. Even though surgery can restore anatomical structure, glial scar formation and lack of neurotrophic factors inhibit axon regeneration, and surgery cannot solve this problem. Moreover, some patients developed postoperative hypertonia or neuropathic pain, requiring long-term drug control.

**HIGHLIGHTS**

- The study comprehensively describes and discusses the functional role of Schwann cell (SC) transplantation in spinal cord injury (SCI) repair.
- The treatment differences of SC transplantation in clinical trials of SCI were discussed over time.
- The current progress and challenges of SC transplantation in clinical trials of SCI were comprehensively analyzed.

Medical treatment includes neurotrophic drugs (such as mecobalamin), hypotonics (such as baclofen and botulinum toxin), neuropathic pain relief drugs (such as gabapentin and pregabalin), subsequent rehabilitation therapy, and/or surgery in conjunction with these treatments. Although the combined use of these methods can accelerate and enhance a patient’s functional recovery, most patients with initial complete injury have severe irreversible neurological deficits and are unable to restore apoptotic/dead nerve cells, rebuild neural network function, and achieve effective treatment^[9]^. In addition, long-term drug treatment has its own limitations, including side effects, truncation, and dependence. Therefore, it is of great significance to find a foreground treatment to improve these changes after SCI and promote the repair and functional recovery of SCI.

With the continuous exploration of SCI treatment, functionally active cells have been widely used in the fields of nerve injury and tissue repair in recent years. People transplant functional active cells (such as olfactory ensheathing cells (OECs), mesenchymal stem cells (MSCs), neural stem cells (NSCs), or induced pluripotent stem cell (iPSC)-derived neural progenitors) into the host to play an important role in promoting tissue damage repair and nerve regeneration^[10,11]^. These functionally active cells play a therapeutic functional role in the treatment of SCI and have demonstrated their application prospects during the clinical trial stage, enhancing the American Spinal Cord Injury Association (ASIA) level of patients with SCI and restoring some motor and sensory functions. These cell therapies also include cell-to-cell combination therapy, cell combination with other therapies (such as biomaterials), and post-cell transplant rehabilitation therapy. These combination therapies improve the effectiveness of cell transplant therapy^[12,13]^. However, these cells differ in functional and biological characteristics. For example, OECs exhibit heterogeneity and plasticity in morphology, antigenicity, and function (the functional heterogeneity of OECs from different sites in nerve injury repair). Genes and proteins differentially expressed by OECs derived from the olfactory bulb play a key role in nerve regeneration, axon regeneration and extension, transmission of nerve impulses, and response to axon injury. Genes and proteins differentially expressed by OECs derived from the olfactory mucosa are mainly involved in the positive regulation of inflammatory responses, defense responses, CK binding, cell migration, and wound healing^[14,15]^. This also leads to differences in the therapeutic effects of OECs derived from different locations on nerve injury. Moreover, the purification steps of OECs are numerous and costly, and the activity is reduced.

MSCs, including bone marrow MSCs (BMSCs), have the characteristics of infinite proliferation and differentiation. MSCs exist in various tissues in adults, such as adipose tissue, nerve tissue, cord blood, and dermis. They are rich in origin and can replicate and regenerate as undifferentiated cells. These cells exhibit a stable phenotype and retain their multi-directional differentiation potential *in vitro*^[16,17]^. However, the survival rate of transplanted MSCs in an ischemic, inflammatory, or hypoxic neural injury environment is significantly reduced (usually <10%). The efficiency of MSC differentiation into functional neurons or glial cells *in vivo* is low. The differentiated cells may lack the electrophysiological functions of mature nerve cells and are difficult to specifically migrate to nerve injury sites^[18,19]^. MSCs may also change from an anti-inflammatory phenotype to a pro-inflammatory phenotype, exacerbating nerve damage^[20]^. NSCs have the potential to self-renew and differentiate into neurons, astrocytes, and oligodendrocytes and can replace lost/apoptotic neurons after SCI^[21]^. NSCs can also support neuron survival and axon regeneration by secreting neurotrophic factors, promoting angiogenesis and immunoregulation^[21]^. Despite these advantages of NSCs, there are still huge obstacles to their use in SCI treatment. NSCs have limited sources (derived from embryonic tissue), and *in vitro* culture is complex and costly, with long cycles and low survival rates. NSCs can be affected by adverse environments such as local hypoxia and inflammation after SCI, resulting in a large number of cell deaths, a significant reduction in survival rate, and differentiation efficiency after transplantation. They may also lead to the formation of non-functional cells (such as scar astrocytes) and even form a tumor-like structure. Allogeneic NSCs, especially human embryonic stem cells, can cause immune rejection and require long-term immune suppression. They are also subject to ethical and moral disputes that limit their clinical application. The culture protocols of NSCs (such as growth factor combinations and differentiation induction conditions) vary greatly in different laboratories, making the efficacy difficult to replicate.

iPSCs make a valuable contribution to regenerative medicine by bypassing the ethical issues of using human embryos. Converting human iPSCs into differentiated derivatives is an efficient and effective method to transform human iPSCs into differentiated derivatives. Although iPSC-derived neural generators can solve many problems of insufficient cell origin, they have also shown a positive role in promoting nerve injury regeneration^[22,23]^. iPSCs can differentiate into a large number of high-purity neural stem/neural progenitor cells, but this process takes a long time, has low survival rates, is cost-effective for developing treatments, and comes with safety issues. The transformation of iPSCs into clinical applications has become complex. Genetic instability/uncertainty during the transformation/differentiation process may lead to the risk of tumorigenesis or malformation.

Compared with these cells, Schwann cells (SCs) have certain advantages and have been extensively studied in nerve injury repair. SCs constitute the glial cells of the myelin sheath of the peripheral nerve and play the role of supporting and nourishing neurons. In the process of nerve injury repair, SCs can transform into a repair phenotype, promoting axonal regeneration and re-myelination. Moreover, SCs recruit macrophages by secreting some chemokines and CKs, promote phagocytosis of apoptotic cells and myelin fragments, and provide the necessary basis for nerve regeneration^[24,25]^. Another characteristic of SCs is that they can transform autophagically, remove myelin sheath, and provide support for repairing nerve damage. SCs can also secrete a variety of different neurotrophic factors, such as nerve growth factor (NGF), brain-derived nerve growth factor (BNDF), neurotrophic factor-3, neurotrophic factor 4/5, fibroblast growth factor, platelet growth factor, and extracellular matrix (ECM), which provides a good basic environment for neuron survival and regeneration. SC transplantation can improve the secondary pathological injury process after SCI, including inhibiting inflammatory cell activation and release of inflammatory CKs, inhibiting glial scar formation, and guiding new axons to cross the scar area to form new connections with distant axons of the host^[26,27]^. These characteristics all demonstrate the wide application value of SCs in the field of nerve regeneration. After transplantation of SCs differentiated from skin progenitor cells into spinal cord contusions in rats, GFP+ cells (labeled as SCs) were present in the spinal cord of all animals. Twenty-one weeks after transplantation, there were 20 000–120 000 GFP+ cells in the SCI area of the animals. It was found that SCs could survive in the host after transplantation, integrate with host tissue, and reduce the formation of dense glial scar^[28]^. The transplanted SCs filled most of the injured sites and greatly increased the survival of endogenous SCs, resulting in the formation of myelin sheath of thousands of germinating host axons around the injured site^[28]^. In addition, the expansion rate, survival rate, and purity of SCs in autologous culture are also high. Studies have shown that SCs cultured from the lower leg and sciatic nerves of 18 clinical trial SCI patients increased in each passage from P0 to P3, with the largest expansion rate between P2 and P3^[29]^. The average production of P2 cells was 8.72 ± 89.2 million cells, and the average production of P3 cells was 15.09 ± 12.99 million cells. In addition, the survival rate and purity of almost all cell products remain above 90%^[29]^. This also reveals that it provides a reliable and compliant method and source for autologous SCs products suitable for regenerative treatment of patients with SCI. All these reveal the functional role of SCs in SCI and suggest that SCs are a promising candidate cell type for the treatment of SCI. Previous studies have reviewed the role of other cell types such as SCs in SCI, including the combination of other cells and substances such as neurotrophins, OECs, steroids, or chondroitinase, providing therapeutic effects of SCs^[12,30]^. However, these lack a detailed summary and discussion of the functional mechanisms of SCs in SCI repair, including promoting axon regeneration and myelination, secreting neurotrophic factors, genetically modifying overexpressing SCs, inhibiting scar formation, and exerting anti-inflammatory and immunoregulatory effects. There is also a lack of new information on SCs in SCI applications, including joint biomaterials, especially in clinical trials in recent years, and the current problems and challenges. Therefore, this article discusses and summarizes these unique characteristics of SCs in a complete and detailed manner. In addition, we summarized the current research status of SCs in clinical trials and deeply analyzed the many problems and challenges existing in SCs in clinical trials of SCI.

## The mechanism of SC transplantation in repairing SCI

### SCs promote axonal regeneration and myelination

The key pathological changes after SCI are neuronal degeneration/apoptosis and axonal retraction, resulting in varying degrees of sensory and motor dysfunction. Therefore, protecting the survival of neurons, reducing their apoptosis, and promoting axon regeneration and re-myelination are very important for the functional recovery of SCI^[23,31]^. An important factor in nerve regeneration is that the regenerated axon must extend through the injured site to the distal end and re-form the nerve connection. Axonal regeneration to an appropriate target is still a challenge, and inappropriate reinnervation is an obstacle to complete recovery^[32,33]^. SCs are plastic in the process of peripheral nerve injury and provide a good basic environment for nerve regeneration. Nerve injury triggers the transformation of myelin and non-myelin SCs into phenotypes that specifically promote repair. At the distal end of the injury, these repaired SCs provide necessary signals and spatial clues for the survival, axonal regeneration, and target nerve reinnervation of the injured neurons^[34]^. Moreover, regenerated axons can be used as a guide for the migration of SCs and maintain the functional and phenotypic changes of SCs^[34]^. It was found that SCs lacked axonal guidance and contact for a long time during nerve injury, and their repair ability decreased^[35]^.

**Figure 1.**
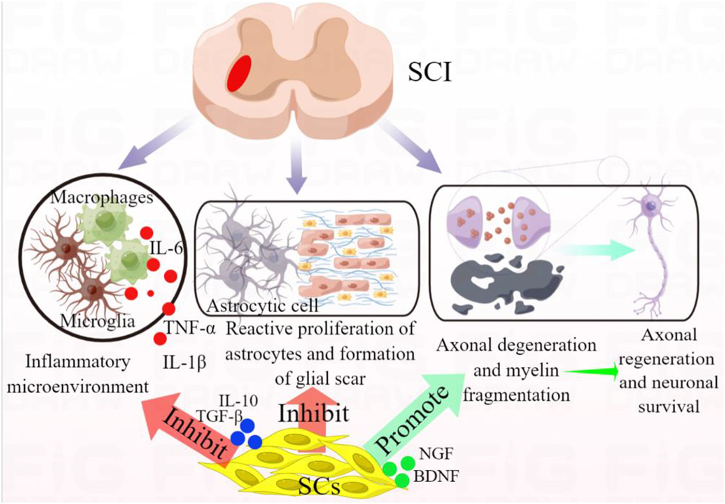
Role of SC transplantation in the repair of SCI.

The important feature of SC transplantation in the treatment of SCI is that it can play a similar role in the repair of peripheral nervous system injury by promoting axonal regeneration and myelination^[36]^ (Fig. 1). Indeed, when SCs are transplanted into the injured spinal cord, they can provide not only a matrix for axonal growth, but also a source of cells for myelin axons. SCs in the dorsal root are reprogrammed to repair SCs even after SCI and then migrate to the injured spinal cord^[17]^. Repaired SCs expressed C-X-C motif chemokine receptor 4 (CXCR4), and its ligand C-X-C motif ligand 12 (CXCL12) was up-regulated in the injured spinal cord. Pharmacological inhibition of CXCR4 signaling reduced penetration of repaired SCs. Moreover, administration of CXCR4 agonists effectively increased penetration of repaired SCs and improved motor function^[37]^. Moreover, SCs can guide the directional extension of newborn axons and establish a new neural network^[38,39]^. Seven days after injecting cultured SCs or SC culture medium into the damaged spinal cord through the subarachnoid space (using titanium clips to crush the spinal cord and establish a rat model of spinal cord contusion), during the tracking process, SCs (labeled with Hoechst) showed their presence in the spinal cord^[40]^. It further showed that the axon density of the injured site was significantly higher 60 days after transplantation, which improved the motor ability of rats^[40]^. Studies have shown that rapamycin can promote autophagy by preserving nerve tissue, increase myelin regeneration mediated by SCs, promote myelin formation, and improve motor function recovery after spinal cord semi-contusion^[41]^. This may be related to the re-myelination of SCs after human SCI. Studies have shown that metformin treatment improves functional recovery by reducing white matter loss and promoting myelination in SCs. The Nrg1/ErbB signaling pathway may be involved in the process of myelination^[39]^. SCs and Matrigel solution were placed in a complete spinal cord transection, improved regeneration of white matter axons across interfaces. The regenerated brainstem axons form synaptophysin terminals and contact the MAP2A (+) dendrites at the caudal interface. Brainstem axon regeneration is directly related to the elongation of glial fibrillary acidic protein (GFAP+) astrocytes into SC bridges and is associated with improved exercise after injury^[42]^. This study determined the nature of the spinal cord/SC bridge interface that allows regenerated brainstem axons to pass through SCI areas, potentially leading to improved motor function that is associated with axon regeneration and functional recovery after human SCI. The chondroitinase ABC gene was transplanted in conjunction with SCs to the spinal cord transection injury site. It was found that SCs migrated over a long distance in the cephalad and caudal directions^[43]^. This migration of SCs leads to enhanced axon regeneration, including adrenergic and dopaminergic axons originating from the supranaspinal region, and promotes restoration of motor and bladder function. Importantly, even after 3 months of treatment simulating human chronic SCI, SC survival and axon regeneration continued until 6 months after injury^[43]^.

Secondary pathological changes after SCI include local inflammatory microenvironmental changes (immune cell activation), reactive astrocyte proliferation, glial scar formation, axonal degeneration/apoptosis, and myelin fragmentation. SC transplantation can improve the inflammatory microenvironment by regulating microglia/macrophage polarization, inhibiting their activity, and the release of inflammatory cytokines. By interacting and integrating with astrocytes, SCs can inhibit astrocyte activation and glial scar formation. Moreover, SCs can also paracrine a variety of neurotrophic factors to protect neural survival, promote axon regeneration and myelin formation, repair SCI, and promote functional recovery.

**Figure 2.**
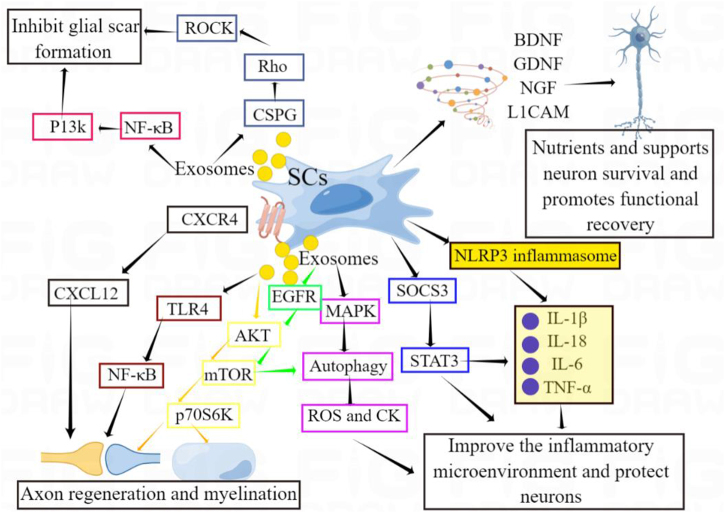
SC transplantation promotes SCI repair by mediating signaling pathways and secreting cytokines.

Exosomes are a kind of vesicles containing a variety of miRNA (such as miR-709, miR-210, and miR-155), mRNA, and proteins^[44–47]^, which are secreted by cells and play an important role in cell-to-cell communication^[48,49]^. Intravenous injection of MSC-derived exosomes reduces the SCI area by regulating Neutrophil Extracellular Trap by exosome miR-125a-3p and significantly improves motor function recovery in rats^[50]^. Other studies have shown that exosomes derived from BMSCs carrying Mir-26a-5p inhibit the expression of EZH2 to promote the expression of BDNF, TrkB, and CREB phosphorylation, and increase the expression of KCC2, thereby protecting PC12 nerve cells and improving SCI^[51]^. SCs can also produce exosomes through a paracrine mechanism. These exosomes are transplanted into the host of SCI, the results similar to that of mother cell transplantation in axonal regeneration and functional recovery are also obtained^[52]^. Exosomes derived from SCs (SC-Exos) and methylprednisolone composite mesh can inhibit neuron death after spinal cord contusion through TLR4/NF-kB, MAPK, and Akt/mTOR pathways, increase neuron survival, and significantly improve the function and electrophysiological properties of SCI rats^[52]^ (Fig. 2). In the model of SCI (establish a spinal cord contusion model by dropping an 8-g striking rod from a height of 4 cm from the T10 spinal cord segment as the center), SC-Exos can be endocytosed by brain-derived endothelial cells.3 (bEnd.3 cells) and promote proliferation, migration, and formation of bEnd.3 tubes. SC-Exos highly expresses the pro-angiogenic molecule integrin-β1 and transplantation into rats can promote angiogenesis in SCI, reduce tissue damage, and improve functional recovery^[53]^. This is related to secondary ischemic pathological changes after human SCI, and SCs can play a role in promoting angiogenesis. Endothelial cell-derived exosomes promote the transformation of the phenotype of SCs in a PI3K/AKT/PTEN-dependent manner and maintain the repair phenotype of SCs, thereby promoting axon regeneration and myelination^[54]^. Extracellular vesicles of SCs derived from skin precursor cells promote axon growth and regeneration of motor neurons by activating the Akt/mTOR/p70S6K signaling pathway, improving the function of spinal cord axotomy injury^[55]^. Exosomes derived from endothelial cells can promote and maintain the repair-related phenotype of SCs, thus promoting axon regeneration, myelin formation of regenerated axons, and recovery of injured nerve function^[54]^. In addition, SC-derived exosomes can increase autophagy and reduce apoptosis after SCI, thus promoting axonal protection and recovery of motor function^[56]^.

SC transplantation can promote axonal regeneration and myelin formation, inhibit inflammatory cell activation, and inhibit glial scar formation through paracrine pathways, including the production of exosomes and neurotrophic factors (such as NGF, GDNF, BDNF, and LICAM). Their role in promoting functional recovery after SCI is achieved through mediating different signaling pathways, including CXCR4/CXCL12, TLR4/NF-kB, EGFR/AKT/mTOR, AKT/mTOR/p70S6K, and MAPK/autophagy/ROS.

In view of the role of SCs in promoting the regeneration of SCI, different studies have used some biomaterials (such as hydrogels or 3D-printed materials) or scaffolds combined with their co-transplantation to enhance the repair of SCI and promote nerve regeneration^[57,58]^. A catheter was inserted into the spinal cord of eight segments of the thoracic vertebra to continuously injure both ends, and SCs were transplanted into the catheter, thus promoting the growth and connection of axons at the interface of the host tissue^[59]^. Although the contusion model is the most commonly used because it is the most common type of SCI in humans. The complete transverse model, while not as clinically significant as the contusion model, is the most rigorous method for assessing axon regeneration. The study reveals that in a complete cross-sectional SCI model, SC transplantation promotes axon growth and crosses the injury area to bridge with host tissue. Studies have shown that SCs can adhere and proliferate when inoculated on fibrous piezoelectric polyvinylidene fluoride trifluoroethylene (PVDF-TrFE) conduit *in vitro*. PVDF-TrFE scaffolds enhanced the functions of SCs to promote axonal growth and regeneration^[60]^. As a kind of bioremediation material, hydrogel has the functions of promoting cell proliferation or drug delivery, anti-inflammatory and immune regulation, and anti-oxidative stress, which provides a good basic environment for SCI repair^[61]^. The structure of 3D-printed materials can provide a favorable microenvironment for the survival, proliferation, and differentiation of SCs in the injured area^[62]^. 3D-printed biomimetic scaffold promotes the formation of connections and targeted cell differentiation between SCs and MSCs during SCI, supports and guides axon growth, and repairs motor function after SCI^[62]^. Activated SCs were encapsulated in 3D GelMA hydrogel and then transplanted into the spinal cord of a half-cut SCI model rat. It was found that SCs showed good survival and proliferation in 3D GelMA hydrogel, inhibited cell death after SCI, significantly reduced spinal cord cavities, and improved functional recovery^[58]^. Decellularized porcine peripheral nerve-based injectable hydrogels enhance the vitality of SCs and improve the therapeutic effect of SCs in treating SCI^[63]^. This also reveals that biomaterials can transport SCs at SCI sites and improve SCI treatment efficacy by maintaining higher SC viability.

### Anti-inflammation and immune regulation of SCs

SCI can initiate secondary cascade injury and lead to ischemia, oxidative injury, edema, and glutamate excitotoxicity. However, the key factor in secondary pathological progress is the change of local inflammatory microenvironment^[64]^. Inflammation in SCI involves many cell groups, such as astrocytes, microglia, T cells, neutrophils, monocytes, acellular mediators, and released inflammatory CKs [such as tumor necrosis factor-α (TNF-α), interleukin-1β (IL-1β), and interleukin-6 (IL-6)] to form a local microenvironment that is not conducive to nerve regeneration^[65,66]^. The activation of immune cells and extensive infiltration are the key factors leading to neuroinflammation. These immune cells are guided to the lesion site by CKs and chemokines released by microglia, astrocytes, and peripheral macrophages in the lesion^[67]^. These can lead to the destruction of the spinal cord blood barrier and edema, excitotoxicity of glutamate, free radicals, and inflammation, which are characterized by demyelination and apoptosis of neuronal tissue^[67,68]^. After injury, the activation and proliferation of glial cells lead to extensive and persistent myelin loss and axonal injury^[69]^.

Currently, a number of anti-inflammatory drugs, such as corticosteroids, nonsteroidal, statins, methylprednisolone, progesterone, and specific CK inhibitors, are used to improve the neuroinflammatory response induced after nerve injury. Although these anti-inflammatory drugs play a role in improving neurological function, results vary depending on the injury model and/or treatment window^[70]^. Importantly, these anti-inflammatory drugs are difficult to reverse secondary pathological changes triggered after nerve injury, fail to repair apoptotic/dead nerve cells, promote axon regeneration, and restore neural networks. Therefore, it is crucial to explore a method that can inhibit the activation and penetration of immune cells, reduce the inflammatory cascade, reverse the catastrophic inflammatory response secondary to nerve injury, provide nerve protection, and enhance nerve regeneration.

One of the important characteristics of SCs is that they have immune activity and play a key role in innate and acquired immune responses. Through interaction with immune cells, SCs contribute to the formation of immune responses that may lead to inflammatory neuropathy^[71,72]^. SCs may regulate local immune response by recognizing and presenting antigens and paracrine production of CKs, or they may influence and stop neuroinflammation by secreting CKs^[73]^ (Fig. 1). The change of inflammatory environment leads to the deficiency of SCs’ response, which is the potential mechanism of nerve regeneration damage. The dedifferentiation phenotype of SCs appears in nerve injury, accompanied by an activation repair program independent of the injury^[74,75]^. The expression of NOD-like receptor protein (NLRP)1 and NLRP3 inflammasome complexes and their related pro-inflammatory CKs increased after SCI. SC transplantation could significantly reduce the expression of these inflammasomes, improve the motor dysfunction, and inhibit nerve cell death and demyelination. It is suggested that SCs may be realized by weakening the activity of the inflammasome complex and related inflammatory circuits^[76]^. Moreover, macrophage/microglia polarization plays an important role in regulating inflammatory response in SCI^[77,78]^. M2 macrophages could be induced 3–7 days after spinal cord contusion (exposed spinal cords by laminectomy at T10, contused the spinal cord with the NYU impactor by dropping a 5-g rod 6.25 mm onto the spinal cord), but M2 markers decreased or disappeared 1 week later^[79]^. The response of M1 macrophages was quickly induced and then maintained in the injured spinal cord^[79]^.

SCs can improve the inflammatory response after SCI by regulating the activity of macrophages/microglia. Acetyl-11-keto-beta-boswellic acid mediates the Nrf2/HO-1/IL-10 signaling pathway to transform macrophages from M1 type to M2 type, reduces inflammation and oxidative stress (OS), and promotes SC migration, thereby accelerating the repair of SCI^[77]^. Transplantation of SCs after macrophage depletion can improve the survival rate of SCs after SCI. Immunofluorescence shows that GFP-labeled SCs in the SCI area are significantly viable and can integrate with the host tissue, reduce the volume of cysts and lesions, and promote functional recovery^[80]^. SC-derived exosomes containing MFG-E8 alter macrophage/microglia polarization by activating the SOCS3/STAT3 pathway to reduce inflammation after SCI. Knockout of MGFG-E8 in SCs could reverse the anti-inflammatory effects of SC-derived exosome therapy. The SOCS3/STAT3 signaling pathway was identified to be involved in upregulating M2 polarization induced by MGFG-E8^[81]^. SC-derived exosomes can effectively mitigate OS and inflammation subsequent to a rat model of compressed SCI, while concurrently diminishing necroptosis. *In vitro* experiments have shown that SC-derived exosomes enhance mitotic autophagy in PC12 cells, leading to reduced production of reactive oxygen species (ROS) and inflammatory CKs triggered by oxygen-glucose deprivation-induced damage. The mechanism may be that SC-Exos promote autophagy by activating the AMPK signaling pathway^[82]^. SC-derived exosomes can decrease the expression level of epidermal growth factor receptor and inhibit the Akt/mTOR signaling pathway, thereby up-regulating the level of autophagy, reducing cell death, exclusively inducing microtubule acetylation and polymerization, and restoring motor function^[56]^.

By inhibiting M1 polarization and stimulating M2 polarization in SCI, SC-Exos can reduce inflammatory response, inhibit neuronal apoptosis, and promote functional recovery in rats^[81]^. Studies have shown that increased macrophages and inflammatory signals reduce the ability of aging nerve regeneration by changing the behavior of SCs^[83]^. SC transplantation significantly reduced the number of microglia/macrophage markers CD11b, CD68, IBA1, and inducible NO synthase-labeled cells in spinal cord contusion, increased the number of anti-inflammatory phenotypes, and inhibited the ability of proinflammatory microglia and macrophages phenotypes^[84]^. When SCs and OECs were transplanted into SCI hosts, it was found that the transplanted cells were more widely distributed, the number of inflammatory astrocytes and microglia/macrophages was reduced, and the expression levels of chemokines CCL2, CCL, IL-6, and TNF-α were reduced. However, the number of anti-inflammatory macrophages/microglia increased, the levels of IL-10 and IL-13 increased, and the cyst size decreased^[85]^. These studies revealed that SCs demonstrated their anti-inflammatory effects in animal models of spinal cord contusion, which simulated secondary inflammatory pathological changes after human spinal cord contusion. SC transplantation provides good support for the repair and functional recovery of SCI by regulating the activity/penetration of immune cells and improving the inflammatory microenvironment.

### SCs inhibit glial scar formation

An important factor in the pathological process of SCI is the formation of glial scars by reactive astrocytes^[86]^. After SCI, it can develop from a small cystic cavitation area to a huge enlarged secondary injury surrounded by glial scar tissue^[87]^. Resident astrocytes and fibroblasts cause permanent glial scars in the injured spinal cord^[86,88]^. Glial scar and its surrounding cell deposition affect axonal growth, including ECM proteins such as chondroitin sulfate proteoglycan (CSPGs), laminin, collagen, and fibronectin, as well as many factors such as transforming growth factor (TGF)-β and transcriptional activator 3^[88,89]^. After SCI, the proliferated astrocytes formed thick cell processes and a network to surround the lesions, and the boundary of glial scar was established. This fibrous scar leads to neurotransmitter disorders, limits angiogenesis, neuronal processes, axonal extension, and extracellular structural reorganization, which is an important obstacle to endogenous repair^[90,91]^. Therefore, the improvement of glial scar is another important factor in repairing SCI.

SCs can improve the reactive proliferation of astrocytes, inhibit the formation of glial scar, and guide the extension of regenerated axons through the scar area. After SCI, myelin fragments can induce astrocyte death *in vitro* and promote the transformation of astrocytes into A1 astrocytes *in vivo*. Astrocytes can swallow myelin fragments through ATP-binding cassette transporter family member A1 (ABCA1). These swallowed cells tend to transform into A1 astrocytes^[92]^. SCs can indirectly reduce A1-type astrocyte activation by paracrining some anti-inflammatory factors such as IL-10 and TGF-β^[84,93]^. Transplantation of SCs can upregulate the expression of the A2 astrocyte marker (S100A10), play a neuroprotective function, and promote tissue repair^[84]^. In addition, SCs can release neurotrophic factors such as BDNF, NGF, and ciliary neurotrophic factor (CNTF) through paracrine pathways and promote A2 polarization by activating Trk receptors on astrocytes^[94,95]^.

SCs with overexpression of miR-124 can migrate actively and enter the astrocyte region, which increases the number of SCs and reduces the expression of GFAP and p-STAT3 (phosphorylated transcription 3) in astrocytes^[96]^. The overexpression of miR-124 in SCs promotes the integration of SCs and astrocytes *in vitro* and may weaken the ability of astrocytes to form a glial scar^[96]^. SC-derived exosomes can increase the expression of TLR2 on astrocytes through the NF-kB/PI3K signaling pathway and reduce the deposition of CSPGs, thereby promoting functional recovery in mice after SCI^[97]^. Further studies also showed elevated TTP-α levels and increased CSPGs deposition during glial scar formation in mice with SCI. After transplantation of SC-derived exosomes, CSPG deposition was reduced in scar tissue, and motor function was restored. The use of Rho/ROCK pathway inhibitors inhibited the repair effect of SC-derived exosomes on scar tissue after SCI^[98]^.

SCs and unidirectional polypropylene filament channels precoated with laminin reduced the astrocyte proliferation at the graft–host boundary^[99]^. Another study showed that SCs were magnetized with superparamagnetic iron oxide nanoparticles coated with poly l-lysine and showed the enhanced migration ability along the force direction. SCs crossed the stellate cell–SC boundary and migrated a longer distance into the stellate cell monolayer^[100]^. It is further demonstrated that polysialyltransferase-functionalized superparamagnetic iron oxide nanoparticles induce overexpression of nerve cell adhesion molecule polysialylation, enhance the migration ability of SCs, increase the number and contact of SCs to astrocytes, cross the boundary of glial cells, and guide the regenerated axons to their distal destination^[101]^. Other studies have found that electroacupuncture can not only promote the survival and proliferation of transplanted SCs but also inhibit the apoptosis of SCs and prevent the formation of astrocytic scar^[102]^. Electroacupuncture can improve the formation of astrocytes by inducing the protuberance of astrocytes at the interface of SC grafts, thereby promoting functional recovery in a rat model of acute SCI^[102]^. These studies show that SCs have the ability to migrate, inhibit glial scar formation, and guide axonal extension by interacting with astrocytes.

### SCs express neurotrophic factors

Another characteristic of SC transplantation in the repair of SCI can provide a good nutritional environment for nerve regeneration and neuronal survival by secreting a variety of neurotrophic factors. Different studies have confirmed that neurotrophic factors play an important role in nerve regeneration. Overexpression of neurotrophic factors can promote regeneration of SCI^[38,103]^. SCs can produce these nutritional factors through paracrine to promote nerve regeneration and functional recovery^[104,105]^. Studies have shown that multiluminal nutrition factor and glial-derived neurotrophic factor (GDNF) have a synergistic effect on promoting sensory nerve axon density and motor axon growth in spinal cord tissue^[106]^. The expression and secretion of progenin granules (PGRN) of SCs depend on the state of cell differentiation. With the dedifferentiation of primary SCs, the expression and secretion of PGRN increased significantly. While cyclic adenosine monophosphate could inhibit the secretion of PGRN and induce the differentiation of SCs^[107]^. In addition, some studies combine loaded neurotrophic factors with transplantation of SCs to enhance the ability of SCs and repair of spinal cord contusion^[108]^. Positively charged polyethylene glycol fumarate is a biodegradable hydrogel for the repair of SCI. By grafting the glial cell-derived neurotrophic factor gel scaffold in conjunction with SCs onto the transection of SCI, the formation of myelin sheath on regenerated axons is increased, the survival and number of neurons are protected, and motor function is partially restored^[109]^. After spinal cord transection, the alginate gel implanted with SCs was transplanted into the injured site, and AAV5 expressing brain-derived neurotrophic factor (BDNF) was injected into the tail of the rat; hence, the number of regenerated axons increased significantly^[110]^. These results suggest that the use of vascular endothelial growth factor and/or platelet growth factor in conjunction with SCs may be beneficial for SCI treatment. In addition, some studies overexpress neurotrophic factors [e.g., neurotrophic factor 3 (NT3) and GDNF] in SCs through gene editing to enhance SC activity and SCI repair capabilities. Studies have shown that when NT3-modified SCs combined with NSCs were transplanted into a transverse SCI in rats, a large number of NT-3-positive SCs and a large number of 5-hydroxytryptamine-, GPT-, and SP-positive nerve fibers were observed in or near the transplant site, protecting the survival of neurons^[111]^. The transplantation of SCs overexpressing GDNF into the lesion space and caudal chord builds a permissive pathway for axon growth and demonstrates that this new permissive bridge promotes the regeneration of descending native spinal tract axons beyond the lesion area. GDNF significantly improves the graft–host interface by promoting the integration between SCs and astrocytes, especially the migration of reactive astrocytes to the SCs-GDNF area. It was found that giant axons regenerated at the lesion and returned to the caudal side of the spinal cord, which improved motor function^[103]^. Studies have shown that transplantation of SCs that overexpress GDNF can guide the growth of axon cut ends to the distal innervation sites of the spinal cord and transcend regeneration between spinal cord hemisection lesions^[112]^. In the distal host spinal cord, the axons of regenerated descending intrinsic spinal cord neurons form synapses with the host neurons, resulting in the restoration of action electricity and partial functional restoration^[112]^. This also means that in preclinical or clinical studies, neurotrophic factors can be overexpressed in SCs through genetic modification or gene transfer to enhance their paracrine functions, provide a nutritional microenvironment, and promote neural network reconstruction in SCI treatment.

It is worth noting that neurotrophic factors can also play the opposite role. Mature BDNF is a member of the neurotrophic factor family, which can inhibit the migration of SCs^[113]^. ProBDNF, the precursor of brain-derived neurotrophic factor, also inhibited the collective migration and chemotaxis of RSC96 cells (a spontaneous immortalized rat SC line) *in vitro*, including inhibition of F-actin polymerization and local adhesion kinetics of cultured RSC96 cells^[113]^. CNTF is highly expressed in SCs and mediates neuroinflammatory response by activating STAT3 signal transduction and inducing the IL-6 release in sensory neurons^[114]^. The application of recombinant CNTF to the sensory nerve can reproduce the neuroinflammatory of the dorsal root ganglion and spinal cord, which leads to pain^[114]^. These studies show the inherent relationship between SCs and neurotrophic factors. Neurotrophic factors play a role in promoting or inhibiting regeneration in SCI. Therefore, we can consider the method of gene modification or transfection in SCs to improve the positive role of neurotrophic factors in promoting regeneration in SCI.

## Clinical trial and current challenge of SC transplantation in SCI

Currently, nerve regeneration and functional recovery of patients can be promoted in a variety of ways, including surgical treatment, electrical stimulation, functional training, and neural rehabilitation^[5–7]^. Although the combination of these methods can improve some of the patients’ dysfunction, most patients with initial complete injury have severe and irreversible neurological impairment. Therefore, it is very important to improve the pathological changes of SCI and promote the recovery of neurological function of patients. The ability of SC transplantation to repair SCI has been confirmed in the field of basic research. This also prompted researchers to explore the rise from basic research to clinical trials. Indeed, different clinical trials have also confirmed the feasibility and safety of transplantation of SCs into patients^[12,115]^ (Table 1).

**Table 1:** Study on SCs in the clinical trial of SCI

Cell source	Type of SCI	Cell dose	Transplant method	Numbers/follow-up time	Therapeutic effect and significance of efficacy	Side effect	References
SCs from autologous sural nerve	Subacute SCI	0.25 × 10^6^ ml, 1 × 10^6^ ml,. 1.5 × 10^6^ ml	Intramedullary injection of SCs	6 patients/1 year	Among them, one patient had two additional sensory points from baseline, and one patient had no numbness or pain in the T2 skin areas of both arms in three places from elbow to armpit. In another patient, motion-evoked potentials could be detected in both legs, which could activate the myoelectric signals in the legs, and AISA changed from A to B. MRI showed that edema around the SCI area was reduced in all patients, and the length and size of the SCI were reduced.	There were no surgical, medical, or neurological complications after transplantation. No adverse events or serious adverse events related to cell therapy were found. Intramedullary transplantation of autologous SCs is feasible and safe.	Anderson *et al* ^[117]^
Isolation and culture of autologous sural nerve SCs	Chronic SCI	5 × 10^6^/ml	The catheter filled with SCs was implanted into the site of SCI by spinal surgery.	4 patients/6 months	One subject’s ASIA rating temporarily changed from B to C due to a short-term change in sensation after transplantation, which led to a one-level increase in the level of damaged nerves. One participant’s motor function improved by 4 points, sensory function improved by 6 points, and the level of nerve injury improved by 1 level. One participant’s right triceps (C7) strength increased from 2 points to 3 points, which persisted through month 6. The volume of the capsule decreased after transplantation.	No serious adverse events related to transplantation were found, but urinary tract infection and skin abrasion were the most common adverse events.	Gant *et al* ^[119]^
Obtained from autologous sural nerve	Traumatic cervical or thoracic spinal cord injury	5 × 10^6^/ml	Intrathecal transplantation	32 patients/6 months	Among them, 29 patients had significant improvements in the maximum detrusor pressure during the filling period, the maximum detrusor pressure at the maximum urinary flow rate, the maximum urinary flow rate, and post-urination residual volume. The number of incontinence episodes in the patient was significantly reduced compared with baseline, and the total quality of life score improved significantly.	No adverse reactions or other related clinical problems were found.	Akhlaghpasand *et al* ^[124]^
Sural nerve transection to obtain SCs	Thoracic and cervical spinal cord injury	1 × 10^6^/ml	Intramedullary injection of SCs suspension	33 patients/2 years	Patient’s ASIA score for the sensation of light touch increased by 5.5 points, while the sensation of light touch improved more significantly in neck lesions. The light touch sensation score for chest lesions increased by 3.1 points, and the acupuncture sensation score increased by 0.9 points. Athletic ASIA scores improved slightly. Six patients had improved feeling of urination, urinary control rate, and feeling of defecation.	No surgery-related deformities were found by MRI. There were no cases of permanent neurodegeneration or any infectious or viral complications, and no new increase in cavity size or abnormal tissue and/or tumor was found.	Saberi *et al* ^[120]^
SCs obtained from sural nerve	Chronic SCI	0.04–0.06 × 10^6^/ml	The microsyringe was injected into the adjacent area of the SCI.	6 patients/5 years	All patients experienced a pattern of sensory and motor recovery along the trunk and limbs from proximal to distal ends. The ASIA and SEARCH exercise scores increased in all patients. Patient’s autonomous function improved earlier than sensory and motor function (improved skin nutrition, reduced spasticity, increased bladder capacity, reduced residual urine volume, and smooth bowel defecation). Over time, sensory and motor functions gradually improved. MRI showed spinal cord softening and cystic volume reduction.	No serious adverse events occurred in all patients.	Zhou *et al* ^[121]^
SCs obtained from sural nerve	Complete SCI	1 × 10^6^/ml	Autologous transplantation through lumbar puncture	6 patients/30 months	In one patient, the ASIA grade was changed from grade A to grade B, and all the indexes of UDS were improved, especially the improvement of bladder compliance, which was consistent with the axonal regeneration seen by MRI. The patient’s motor score did not improve. Another patient scored 48 for acupuncture and light touch, and felt sensations were detected in the perianal area.	There is no evidence of tumor tissue overgrowth.	Oraee-Yazdani *et al* ^[122]^
SCs obtained from sural nerve	Complete SCI	50 × 10^6^/ml	Intrathecal autologous Schwann cell transplantation	11 patients/12 months	Patient’s SCIM III total score and all subscores included significant improvements in respiratory and sphincter management, mobility, and self-care, with the most significant positive subjective improvements being trunk movement, standing/sitting balance, feeling of bladder and rectal filling, and the ability to urinate spontaneously.	Safety assessment showed no systemic complications, and radiography showed no tumor overgrowth, syringomyelia, or pseudomeningocele.	Oraee-Yazdani *et al* ^[123]^

SCI can be divided into five levels according to the American Spinal Cord Injury Association (ASIA). Grade A is a complete injury: no sensory or motor function retention in the sacral segment (S4-S5) (no anal sensation and spontaneous anal contraction). Grade B is an incomplete sensory impairment: the sacral segment retains sensory sensation (such as anal or deep anal pressure sensation), but no motor function. Grade C is an incomplete sports injury: sacral segment retains motor function (e.g., anal sphincter can contract autonomously), and more than half of the key muscle strength below the injury level is <grade 3 (unable to resist gravity). Grade D is an incomplete sports injury: at least half of the key muscle strength below the injury plane is ≥grade 3 (resistant to gravity).

Grade E is normal, with normal movements and sensations.

Sensory check-inspection site: 28 skin segments (C2-S5, needle stick sensation and light touch on each side). Scoring criteria: 0, missing; 1, diminished or abnormal; 2, normal.

Exercise examination: key muscle groups, 10 groups of muscles (C5-T1, L2-S1), the muscle strength of each side is scored on a scale of 0–5.

At present, SCs with high activity and purity can be obtained from autologous or allogeneic. In the process of passage *in vitro*, the activity and purity of SCs were kept above 90%, which improved the cell foundation for clinical trials^[29,116]^. A phase I clinical trial was conducted to evaluate the safety of autologous SC transplantation to the injury center in six patients with subacute SCI. Autologous SCs from the sural nerve of each participant were injected into the center of SCI. After a year of follow-up, no adverse or serious adverse events related to cell therapy were found^[117]^. Neurological functional changes were assessed using the International Standards for Neurological Classification of Spinal Cord Injury (ISNCSCI) and the ASIA. Two patients added two sensory points from baseline. One patient had no numbness or pain in the T2 skin areas of both arms in three places from elbow to armpit. MRI showed that edema around the SCI area was reduced in all patients, and the length and size of the SCI were reduced. Motor evoked potentials could be detected in both legs of a patient, and AISA changed from A to B. There is no evidence of additional SCI, mass damage, or syringomyelia formation. No surgical, medical, or neurological complications were found, indicating that the cell transplantation procedure is safe^[117]^. Autologous SC transplantation was performed in four patients with chronic SCI. After a 1-year follow-up, they were evaluated according to the ASIA criteria, sphincter, sexual function, and MRI. No adverse reactions, infection metastasis, neurological deterioration, or other related clinical problems were found in any patients^[118]^. One patient with incomplete SCI showed improvement in movement and sensation^[118]^. All four patients experienced a transient increase in sexual function and muscle spasm after transplantation^[118]^. In further clinical trials, autologous SCs were injected into four patients with chronic SCI. No serious adverse events related to SC transplantation were reported. One subject’s ASIA rating temporarily changed from B to C due to a short-term change in sensation after transplantation, which led to a one-level increase in the level of damaged nerves. One participant had an increase in right triceps (C7) strength from 2 points to 3 points, which persisted through month 6. Another participant’s left triceps strength increased from 3 points to 4 points, and this condition remained unchanged in the sixth month^[119]^. One participant’s motor function improved by 4 points, sensory function improved by 6 points, and nerve injury improved by 1 level^[119]^. Follow-up MRI of neck (6 months) and chest (24 months) showed that the volume of cystic cavity decreased after transplantation, and the effect decreased with the passage of time^[119]^. A clinical trial reported that 33 patients received autologous SC suspension transplantation. After 2 years of follow-up, the patient’s ASIA score for light touch sensation increased by 5.5 points, and the improvement in light touch sensation was more significant in neck lesions^[120]^. Light touch sensation scores for chest lesions increased by 3.1 points, acupuncture sensation scores increased by 0.9 points, and exercise ASIA scores improved slightly. Six patients improved their feeling of urination, their urinary control rate, and feeling of defecation. MRI did not reveal any procedure-related malformations. There were no cases of permanent neurological deterioration or any infectious or neoplastic formation^[120]^. Six SCI patients who received autologous SC transplantation were followed up for more than 5 years. All patients developed a pattern of sensory and motor recovery from proximal to distal ends along the trunk and limbs. The ASIA scale and SEARCH exercise scores increased in all patients^[121]^. The patient’s autonomic function improved (improved skin nutrition, reduced spasticity, increased bladder capacity, reduced residual urine volume, and smoother bowel defecation), and this improvement occurred earlier than sensory and motor function. Over time, sensory and motor functions gradually improved^[121]^. MRI showed that myelopathy and cystic lesions became smaller in size after transplantation, and no development of glial tumors, masses, new bleeding, swelling, cyst expansion, new cyst formation, infection, or damage to neural structures was found^[121]^.

In addition, a few clinical trials have produced synergistic therapeutic effects by transplanting SCs in conjunction with MSCs. Six subjects with complete SCI received autologous SCs combined with MSC transplantation. After 30 months of follow-up, one of the patients’ ASIA grade was changed from grade A to grade B, and all indexes of the urodynamic study (UDS) were improved – especially bladder compliance, which was consistent with the axonal regeneration observed on MRI^[122]^. Another patient scored 48 for acupuncture and light touch. For approximately 3 years, the patient’s electromyography showed an increase in the minimum spontaneous motor unit action potential of the anal sphincter^[122]^. UDS showed a feeling of bladder fullness, good compliance and volume, with very slight detrusor overactivity. Magnetic resonance fiberoptic imaging showed axon regeneration in the damaged area^[122]^. A study reported on 11 patients with complete SCI who received a combination of intrathecal autologous MSCs and SCs. During a follow-up period of 12 months, patients’ Spinal Cord Independence Measure III (SCIM III) total score and all subscores, including respiratory and sphincter management, mobility, and self-care, improved significantly^[123]^. The most significant positive subjective improvements were trunk movement, standing/sitting balance, feeling of bladder and rectal filling, and the ability to urinate spontaneously^[123]^. The study’s safety assessment showed no systemic complications, and MRI showed no tumor growth and syringomyelia.

Recently, a randomized phase II clinical trial was reported. Thirty-two patients with complete SCI received intrathecal transplantation of autologous SCs and MSCs. After 6 months, 29 of them had significant improvements in maximum detrusor pressure during the filling period, maximum detrusor pressure at maximum urinary flow rate, maximum urinary flow rate, and post-urination residual volume^[124]^. The number of incontinence episodes in the patient was significantly reduced compared with baseline, and the total quality of life score was significantly improved^[124]^. This study revealed that the combination of MSC and SC intrathecal administration significantly improved the UDS parameters, urinary incontinence rate, and quality of life of patients. Another phase 2 randomized clinical trial reported that transplanting SCs in combination with bone marrow-derived MSCs into 67 patients with complete SCI induced neuropathic pain. After 6 months of follow-up, patients’ pain interference items, including pain frequency, current pain intensity, pain score, and worst numerical rating scale pain intensity scores, showed significant reductions^[125]^. It suggests the effectiveness of combination cell therapy in improving neuropathic pain and quality of life in patients with complete SCI. These studies have revealed the safety and feasibility of SCs in clinical trials for SCI and have a therapeutic effect in promoting functional recovery in patients after SCI. However, these studies have certain limitations. The follow-up period of most clinical trials in these studies currently ranges from 1 to 2 years. There is a lack of long-term follow-up, and there is a lack of evaluation data on long-term treatment effects and long-term safety, including potential risks of tumor formation, and a lack of data to support the long-term functional maintenance of SC transplantation. Moreover, the clinical sample sizes currently used in these studies are small and uneven, and the clinical patient sample sizes used are limited. In addition, large differences in the type of injury (e.g., complete/incomplete) and anatomical location (such as neck/chest) of SCI patients used have resulted in differences in treatment outcomes obtained in existing studies. Therefore, more clinical data and clinical sample sizes, as well as standardization of injury models (such as unified use of chronic intact thoracic SCI), are needed to further support the exact effect of SC transplantation in treating SCI.

Although cell transplantation is a process of continuous evolution and development, which has attracted extensive attention, there are still challenges in clinical trials, which may be related to many problems that need to be solved. First, due to anatomical and biological differences between species, the results obtained in basic animal experiments cannot be directly extended to human trials. The therapeutic effect of SCs in clinical application is not ideal compared with animal injury models. This may be very different from the tailor-made experimental injury model, cell transplantation treatment method, and human primary/secondary SCI, including anatomical and behavioral differences. Rodent SCI models cannot fully simulate human pathology, and large animal models (such as pigs and monkeys) need to be combined to assess motor function recovery and safety. Changes in long-term survival and inflammatory response in immunodeficient or humanized mouse models of allogeneic SCs also need to be evaluated. In addition, SCs may promote the formation of non-functional nerve synapses, lead to neuropathic pain or abnormal autonomic reflexes, and require transcriptome/proteomic analysis, single cell sequencing of SCs homogeneity, and CRISPR editing to optimize cellular function to better understand their dynamic response to injury. Moreover, more basic research is needed to fully understand how SCs work in the body and how they interact with the host, so as to better transition to clinical trials.

Second, the transformation from laboratory research to commercial products faces multiple challenges, including GMP production, safety testing, and regulatory approval. GMP production challenges include issues such as cell origin and standardization, cell purity and consistency, large-scale expansion, and quality control. The problems of cell source, instability (long-term passage may lead to genetic instability, and early generations need to be set as the production endpoint), funding, and logistics have led to an obvious lack of large-scale II and III phase trials of SCs in clinical trials. Moreover, most studies on SCs come from autologous nerves and are obtained by culture *in vitro*, but the imperfection of the culture system *in vitro* and the instability of cells in the culture process can lead to the limitation of clinical trials and differences in therapeutic effects. Autologous SCs can avoid immune rejection, but the expansion cycle is long and the cost is high. Autologous SCs are taken from the patient’s peripheral nerves and require additional surgery to collect materials, which may cause neuropathy at the donor site (such as paresthesia). Allogeneic SCs need to address issues of immune compatibility and donor screening, and long-term use of immunosuppressants (such as cyclosporine), which may increase the risk of infection and tumors. During the acquisition and culture of SCs, strict phenotypic markers (such as S100 and GFAP) must be established. Cell sources with purity standards (e.g., >95%) must be obtained, and strict quality controls, including viability (e.g., >90%), sterility, mycoplasma-free status, and tumorigenicity testing, must be established. Tumorigenicity can be reduced through flow sorting or gene editing (such as CRISPR knocking out proliferating genes). Moreover, in terms of regulatory approval, classification and path selection can be carried out, including ATMP classification and DA/EMA requirements for cell therapies, somatic cell therapy products (SMP), and biological products (BLA path), thereby accelerating the approval process. Furthermore, optimize clinical plan design and implement phased goals – Phase 1: focus on safety (such as adverse events and immune responses) and Phase 2: effectiveness assessed in conjunction with ASIA scores, electrophysiology, or MRI imaging. The CMC (chemistry, manufacturing, and control) document includes process validation and requires three batches of consistency data, including cell bank establishment (MCB/WCB) and stability studies (e.g., post-freeze preservation and post-recovery viability). Process changes need to prove that cell characteristics remain unchanged (e.g., transcriptome analysis).

Third, the best time of cell transplantation, cell dose, and the way or technical problems of cell transplantation. At present, there is no unified optimal time for cell transplantation in many studies. As SCI can be accompanied by secondary injury, the best time for treatment may be missed. Transplantation may lead to cell death due to inflammatory environment in the acute phase, while in the chronic phase, it faces scar barriers, and the optimal time window is not yet clear. Moreover, the dosage and methods of cell transplantation include intrathecal transplantation, intraspinal transplantation, venous transplantation, and local transplantation. The cells transplanted through these methods cannot be colonized at the injured site. For example, intrathecal and intraspinal grafts are invasive procedures that risk damaging nerves, blood vessels, or spinal cord tissue and may lead to pain, bleeding, or infection. Precise positioning is relatively difficult, and cell transplantation requires high targeting. In particular, intraspinal injection needs to avoid damaging key neural structures, which is technically difficult. Transplanted cells may spread to non-target areas, causing side effects (such as ectopic tissue formation). Moreover, repeated treatment of these transplant methods is difficult, and multiple transplants may increase the risk of complications. Recently, scaffold-assisted delivery systems have certain advantages, including providing physical support for transplanted cells, delivering multiple therapeutic components (e.g., scaffold can be loaded with growth factors, anti-inflammatory drugs, or genetically modified cells to achieve collaborative therapy), slowly releasing drugs or nutritional factors to prolong the treatment effect, and implementing controlled cell distribution and directed growth. However, there are also some issues, including surgical invasiveness and implantation risks, increased risk of secondary injury, scaffold degradation and long-term safety issues, cell-scaffold integration issues, individualized adaptation and manufacturing challenges, and clinical transformation obstacles. In addition, the transplant procedure may temporarily activate microglia, release pro-inflammatory factors (such as TNF-α and IL-1β), and exacerbate secondary damage. At present, there is no good method for location and tracking *in vivo*, and it is difficult to evaluate the survival rate and number of transplanted cells *in vivo*, these factors can lead to differences in therapeutic effects, or even failure.

Fourth, different studies have used combined cell therapy methods to produce synergistic therapeutic effects in the treatment of SCI, such as MSCs and oligodendrocyte precursor cells^[126,127]^. Combination treatment can better and effectively promote functional recovery after SCI, reduce glial scar formation, promote angiogenesis, and re-myelinate damaged axons, which has the advantages of overcoming the limitations of single therapy^[126,128]^. However, the application of combined cell therapy still has shortcomings. The inherent risks of some cells may outweigh their potential benefits. For example, cells and tissues with high proliferation potential are at risk of tumor formation. Non-autologous cells may trigger an immune response, which may induce immune overactivation and graft-versus-host disease. Moreover, different cell sources pose great difficulties in terms of technology and standardization. For example, the high cost of cell preparation, quality inspection, and joint application far exceeds that of single-cell therapy. In addition, different cell types play different roles in SCI treatment due to their unique properties, resulting in different results after transplantation and large differences in treatment effects.

In recent years, the activity and function of SCs have been enhanced by combining biomaterial methods (such as hydrogels, scaffolds, or 3D-printed materials), which promote functional recovery from SCI. Biomaterials can provide physical support, fill the space for SCI, prevent glial scars from hindering regeneration, reduce the killing effect of local inflammatory reactions on transplanted cells, and extend SC activity. In addition, biomaterials can also mimic extracellular matrices to enhance SCs’ adhesion and migration (such as RGD peptide repair scaffolds)^[129–131]^. Although SCs combined with biomaterials have these advantages, the combined application does not seem feasible in short-term clinical trials. Due to the mismatch between the effectiveness and safety of the combined graft, including the selection and optimization of biomaterials, the performance of bioactive materials, and the degradation rate (e.g., too fast degradation (collagen) or too slow degradation (PCL) affects repair), mechanical properties (the spinal cord needs to withstand mechanical pressure, and the material needs to be both flexible and strong), and the interaction and functional mechanism of SCs and biomaterials *in vivo*.

In addition, in addition to these methods of treatment, supplementary treatment after cell transplantation, especially post-transplantation rehabilitation programs including electrical stimulation and exercise training, contributes to the recovery of the nervous system and functions. Electrical stimulation is widely used to promote neural activity. Electrical stimulation of the demyelinated area can save local demyelinated axons from conduction failures, activate, and restore axon conductance^[132]^. Electropuncture can alter astrocyte proliferation by inducing the extension of astrocyte processes at the SC graft interface to promote axon regeneration after SC graft. The combination of SC transplantation and electroacupuncture can enhance corticospinal tract axon regeneration and myelination after SCI by upregulating neuregulin 1 type III and downstream signal regulation in SCs^[102]^. This also provides an additional treatment strategy for the treatment of SCI.

Finally, there are other issues, such as the paracrine effect of SCs and their impact on functional recovery, the impact of SCs on the microenvironment of patients with SCI, and the ability of SCs to regulate nerve cell plasticity and rebuild connections. Moreover, there is a certain difference between the functional mechanism of SCs in the repair of central nervous system and the repair mechanism they play in the repair of peripheral nerve injury, which also causes their functional role in promoting the repair of central nerve injury to be inferior to that of the peripheral nervous system. SCs undergo dedifferentiation after the axial process is ruptured after the repair of peripheral nerve injury, proliferate, and arrange into “Büngner” bands, which provide a physical guide channel for regenerated axons^[133,134]^. However, in central nerve injury, there is a lack of a channel for SCs to migrate naturally, and there is no “Büngner” band structure. It is difficult to arrange transplanted SCs in an orderly manner, and the efficiency of myelination is low. Moreover, surface molecules of SCs (such as L1CAM and N-cadherin) can directly interact with growth cones in peripheral nerve injury, accurately guiding axons to extend to target organs. In addition, SCs promote anti-inflammatory macrophage infiltration by releasing chemokines and inflammatory CKs, clearing degenerative debris without exacerbating inflammation^[135]^. During the repair of the central nervous system, astrocytes proliferate to form dense scars, rich in inhibitory molecules such as CSPGs, which can hinder the migration of SCs and axon extension^[136]^. Neurotrophic factors secreted by SCs in the microenvironment of central nervous system injury promote axon growth, but cannot completely overcome the inhibition of regeneration of central nervous system injury. In addition, incomplete immune immunity characteristics of the central nervous system and slow blood supply in the injured area lead to hypoxia/apoptosis of transplanted SCs and reduced survival rate. These may lead to differences in the therapeutic effectiveness of SCs in the repair of central nervous system injuries, including SCI.

It is worth mentioning that emerging evidence suggests that metabolic changes, including mitochondrial dysfunction and changes in glucose and lipid metabolism, contribute significantly to the pathogenesis of nerve damage repair. Mitochondrial dysfunction has been observed in both immune cells and oligodendrocytes during central nervous injury. Impaired mitochondrial function leads to energy shortages that affect key processes such as pulse transmission and axon transport, ultimately leading to neurodegeneration. Mitochondrial dysfunction is associated with the production of ROS, which exacerbates myelin damage and inflammation^[137]^. Glucose metabolism affects oligodendrocyte function and the energy supply required for myelin synthesis. Dysregulation of lipid metabolism leads to changes in the composition of the myelin sheath and affects its stability and integrity^[137]^. Harmful changes in lipid metabolism and excessive accumulation of lipids lead to a lack of axon regeneration, poor recovery of neurological function, and complications after various central nervous system trauma, including brain and SCI^[138]^. This also reflects the conditions under which rewiring lipid metabolism can manipulate therapeutic gains, because it favors axon regeneration and central nervous system repair. SCs can participate in nerve injury repair by regulating energy metabolism^[139,140]^. SCs protect damaged axons through sharp up-regulation of glycolysis due to inherent adaptation to axonal injury. This glycolytic reaction, combined with enhanced axon-glial metabolic coupling, supports axon survival^[141]^. Research shows that the integration of leptin receptor signaling in SCs ensures effective nerve repair by regulating injury-specific catabolic processes in regenerative nerves, including autophagy and mitochondrial respiration^[142]^. These further demonstrate that regulating the ability of SCs to participate in metabolic processes contributes to the neural damage repair process. Therefore, a thorough and full understanding of how SCs affect the detailed metabolic mechanisms of neural injury repair may bring new strategies and insights to the development of neural injury treatments.

## Conclusion

The pathological process of SCI is complicated, which brings great psychosomatic damage to the patients. Currently, there are no effective treatment methods. As a special kind of glial cells, SCs have the characteristics of repair and play a therapeutic role in nerve regeneration. The therapeutic mechanism of SC transplantation in SCI includes promoting axon regeneration and remyelination, inhibiting astrocyte activation and glial scar formation, and guiding regenerated axons to reach the target connection through glial scar. SCs can also improve the local inflammatory microenvironment around SCI, inhibit the activation and infiltration of immune cells and the release of inflammatory CKs, protect neuronal survival, reduce neuroinflammatory response, and promote functional recovery. In the field of basic research and application, the effect of SC transplantation in the treatment of SCI has been affirmed. However, in the current clinical trial stage, SC transplantation into patients has reliable safety and feasibility. SCs are in the initial stage of the treatment of SCI and are challenged and hindered by many problems, but with the passage of time, these problems can be solved step by step. In short, SCs are a promising candidate for treatment.

## Data Availability

All data generated or analyzed during this study are included in this article. We have not used other data that have already been published. All the data presented in this article are original results derived from this study.References
